# Increasing access to CBT for psychosis patients: a feasibility, randomised controlled trial evaluating brief, targeted CBT for distressing voices delivered by assistant psychologists (GiVE2)

**DOI:** 10.1186/s13063-020-4181-y

**Published:** 2020-04-01

**Authors:** Mark Hayward, Clio Berry, Ben Cameron, Kate Arnold, Katherine Berry, Stephen Bremner, Kate Cavanagh, David Fowler, Heather Gage, Kathryn Greenwood, Cassie Hazell, Anna-Marie Jones, Sam Robertson, Clara Strauss

**Affiliations:** 1 School of Psychology, University of Sussex, Brighton, BN1 9RH UK; 2 Research and Development Department, Sussex Partnership NHS Foundation Trust, Hove, BN3 7HZ UK; 3 Research and Development Department, Pennine Care NHS Foundation Trust, 225 Old St, Ashton-under-Lyne, OL6 7SR UK; 4 Faculty of Biology, Medicine and Health, University of Manchester, Manchester, M13 9PL UK; 5 Brighton and Sussex Medical School, University of Sussex, Brighton, BN1 9RH UK; 6 School of Biosciences and Medicine, University of Surrey, Guildford, GU2 7XH UK; 7 Social Sciences Department, University of Westminster, London, W1W 6UW UK; 8 University of Sussex and Sussex Partnership NHS Foundation Trust, Brighton, BN3 7HZ UK

**Keywords:** Self-help, Voice-hearing, Psychosis, CBT, Supportive counselling

## Abstract

**Background:**

The National Institute for Health and Care Excellence (NICE) recommends that Cognitive Behaviour Therapy for psychosis (CBTp) is offered to all patients with a psychosis diagnosis. However, only a minority of psychosis patients in England and Wales are offered CBTp. This is attributable, in part, to the resource-intensive nature of CBTp. One response to this problem has been the development of CBTp in brief formats that are targeted at a single symptom and the mechanisms that maintain distress. We have developed a brief form of CBTp for distressing voices and reported preliminary evidence for its effectiveness when delivered by highly trained therapists (clinical psychologists). This study will investigate the delivery of this intervention by a cost-effective workforce of assistant psychologists following a brief training and evaluate the acceptability and feasibility of conducting a future, definitive, randomised controlled trial (RCT).

**Methods:**

This is a feasibility study for a pragmatic, three-arm, parallel-group, superiority 1:1:1 RCT comparing a Guided self-help CBT intervention for voices and treatment as usual (GiVE) to Supportive Counselling and treatment as usual (SC) to treatment as usual alone (TAU), recruiting across two sites, with blinded post-treatment and follow-up assessments. A process evaluation will quantitatively and qualitatively explore stakeholder experience.

**Discussion:**

Expected outcomes will include an assessment of the feasibility of conducting a definitive RCT, and data to inform the calculation of its sample size. If evidence from a subsequent, fully powered RCT suggests that GiVE is clinically and cost-effective when delivered by briefly trained assistant psychologists, CBTp offered in these less resource-intensive forms has the potential to generate benefits for individual patients (reduced distress, enhanced recovery and enhanced quality of life), service-level patient benefit (increased access to evidence-based psychological therapies) and economic benefits to the NHS (in terms of the reduced use of mental health inpatient services).

**Trial registration:**

Current Controlled Trials, ISRCTN registration number: 16166070. Registered on 5 February 2019.

## Administrative information

**Table:** 

Title {1}	Increasing access to CBT for psychosis patients: a feasibility, randomized controlled trial evaluating brief, targeted CBT for distressing voices delivered by Assistant Psychologists (GiVE2)
Trial registration {2a and 2b}	Current Controlled Trials ISRCTN registration number: 16166070. Registered on 05 February 2019. http://www.isrctn.com/ISRCTN16166070
Protocol version {3}	Version 1, dated 05/11/18
Funding {4}	This project is funded by the National Institute for Health Research for Patient Benefit (project number PB-PG-0317-20029). The views expressed in this publication are those of the authors and not necessarily those of the NHS, NIHR or the Department of Health.
Author details {5a}	Mark Hayward (corresponding author) School of Psychology, University of Sussex, Brighton BN1 9RH & Research & Development Department, Sussex Partnership NHS Foundation Trust, Hove, BN3 7HZ m.i.hayward@sussex.ac.uk Clio Berry School of Psychology, University of Sussex, Brighton BN1 9RH c.berry@sussex.ac.uk Ben Cameron Research & Development Department, Pennine Care NHS Foundation Trust, 225 Old St, Ashton-under-Lyne OL6 7SR Ben.cameron@nhs.net Kate Arnold Research & Development Department, Sussex Partnership NHS Foundation Trust, Hove, BN3 7HZ Kate.arnold2@sussexpartnership.nhs.uk Katherine Berry Faculty of Biology, Medicine & Health, University of Manchester, M13 9PL Katherine.berry@manchester.ac.uk Stephen Bremner Brighton & Sussex Medical School, University of Sussex, Brighton BN1 9RH s.bremner@sussex.ac.uk Kate Cavanagh School of Psychology, University of Sussex, Brighton BN1 9RH k.cavanagh@sussex.ac.uk David Fowler School of Psychology, University of Sussex, Brighton BN1 9RH D.Fowler@sussex.ac.uk Heather Gage School of Biosciences & Medicine, University of Surrey GU27XH h.gage@surrey.ac.uk Kathryn Greenwood School of Psychology, University of Sussex, Brighton BN1 9RH k.e.greenwood@sussex.ac.uk Cassie Hazell Social Sciences Department, University of Westminster, London W1W 6UW c.hazell@westminster.ac.uk Anna-Marie Jones Research & Development Department, Sussex Partnership NHS Foundation Trust, Hove, BN3 7HZ Anna-marie.jones@sussexpartnership.nhs.uk Sam Robertson Research & Development Department, Sussex Partnership NHS Foundation Trust, Hove, BN3 7HZ Sam.robertson@sussexpartnership.nhs.uk Clara Strauss University of Sussex & Sussex Partnership NHS Foundation Trust, BN3 7HZ c.y.strauss@sussex.ac.uk
Name and contact information for the trial sponsor {5b}	Sussex Partnership NHS Foundation Trust, Millview, Nevill Avenue, Hove BN3 7HZ – researchgovernance@sussexpartnership.nhs.uk^1^
Role of sponsor {5c}	The study is sponsored by Sussex Partnership NHS Foundation Trust who will provide at least annual monitoring and audit of the trial and governance procedures. The sponsor played no part in study design; collection, management, analysis, and interpretation of data; writing of the report; and the decision to submit the report for publication

## Introduction {6a}

Cognitive Behaviour Therapy for psychosis (CBTp) has robust evidence for clinical [1–3] and cost-effectiveness [4] and is recommended by the National Institute for Health and Care Excellence (NICE) to promote the recovery of psychosis patients [4]. However, implementation in the UK is extremely poor [5], with only 26% of psychosis patients being offered CBTp [6]. The most consistently reported barrier to implementation is insufficient resources, including a lack of trained therapists with dedicated time to deliver CBTp [7].

The Department of Health’s Forward View for Mental Health [8] and Long Term Plan [9] include a commitment to increase access to evidence-based psychological therapies for patients with psychosis. However, future investments in additional trained therapists with dedicated time to deliver CBTp are unlikely to be made on a large enough scale to significantly and comprehensively increase access to CBTp in the near future. Consequently, additional strategies for increasing access may be required, including a reduction in the resources required to deliver CBTp.

NICE [4] recommend research into two issues that could potentially reduce the resources required to deliver CBTp. Firstly, the duration of CBTp – how many sessions are required to generate sufficient benefit? Our meta-analysis of 10 controlled studies indicated that CBTp delivered over less than the recommended 16 sessions is effective in reducing psychosis symptoms when delivered by trained therapists (typically clinical psychologists) [10]. This focus upon shorter forms of CBTp has recently been taken forward through the development of single-symptom therapies [11] which target only one specific psychotic symptom, with some promising early results [12–15]. Sepsychosis patients and the NHS.condly, the ability of ‘briefly trained’ practitioners to deliver CBTp – can CBTp be delivered by a more cost effective alternative workforce (i.e. not highly trained therapists)? Evidence to date suggests that case managers cannot fulfil this role as outcomes from trials have been inconclusive [16–19] and there are competing demands upon their time [18, 19]. Psychology graduates, however, are well-positioned to undertake such a role [18, 20] as there is a large number of psychology graduates in the UK, the USA and elsewhere; their degree provides them with training in psychological models of emotion and behaviour; and they can be readily employed in assistant psychologist (AP) roles at lower cost than more specialist therapists.

Our response to the questions posed by NICE was to develop a brief form of CBTp targeted at distressing voices. We collaborated with Lived Experience Consultants to turn our self-help CBT book (Overcoming Distressing Voices [21]) into a Guided self-help CBT intervention package (GiVE) supported by a workbook. Our first step was to demonstrate the validity and viability of the GiVE intervention package, confirming that it can be delivered by expert therapists with benefits for patients. We conducted a pilot randomised controlled trial (RCT) of GiVE compared to usual care (*N* = 28) [22]. Results were encouraging with high levels of retention within the study (96%), and a very large between-groups effect for the pre-determined primary outcome of negative voice impact (*d* = 1.78) [23].

Our second step within this current study will be to assess the feasibility of conducting a RCT to evaluate the outcomes of GiVE when it is delivered by briefly trained APs. If found to be feasible, the definitive RCT will form the third step within our programme of research. If GiVE is found to be clinically and cost-effective in a future definitive RCT when delivered by APs, it could substantially increase the number of psychosis patients who are able to access CBTp, which will generate benefits for psychosis patients and the NHS.

## Objectives {7}

The long-term aim of this programme of research is to increase access to CBTp for psychosis patients. This aim will be achieved if a brief and targeted CBTp intervention delivered by a less costly workforce of briefly trained APs is found to be clinically and cost-effective.

The specific aim of the current study is to explore the feasibility of conducting a three-arm RCT of GiVE delivered by APs. A process evaluation, using mixed methods and drawing on Normalisation Process Theory [24], will be used to explore mechanisms and contextual factors that may affect the future uptake and implementation of the intervention, in both research and clinical contexts.

Specifically, this feasibility study aims to assess: (1) the acceptability of the brief and targeted intervention to clinicians (will they refer patients to the study?); (2) the acceptability of the brief and targeted intervention to patients (can patients be recruited and retained, and what are their experiences of the intervention?); and (3) the ability of APs to adhere to the therapy and clinical supervision protocols. We will also estimate the standard deviation of outcomes in order to facilitate a sample size calculation for use within a definitive trial.

## Trial design {8}

This is a feasibility RCT with a three-arm, parallel-group, superiority 1:1:1 allocation, comparing a Guided self-help CBT intervention for Voassessments will be conductedicEs and treatment as usual (GiVE) to Supportive Counselling and treatment as usual (SC) to treatment as usual alone (TAU), recruiting across two sites, with blinded post-treatment and follow-up assessments. Outcomes will be assessed at baseline (pre-randomisation – Time 0), 16 weeks (post intervention – Time 1) and 28 weeks^1^ (follow-up – Time 2). Time 1 and 2 assessments will be conducted by a researcher who is blind to group allocation. Adherence to the therapy protocol will be assessed by APs after each session. AP competence will be assessed by independent raters reviewing a random selection of early, middle- and late-recorded sessions.

A mixed-methods process evaluation drawing upon Normalisation Process Theory [24] will be used to capture participants’, clinicians’ and APs’ experiences of the study and the interventions (GiVE and SC), and their views on the facilitators and barriers to the implementation of GiVE within routine psychosis care pathways. The results will be used to develop logic models for a future definitive trial and for NHS implementation.

## Methods: participants, interventions and outcomes

### Study setting {9}

Participants will be recruited from two sites within the UK National Health Service (NHS) – Sussex Partnership NHS Foundation Trust and Pennine Care NHS Foundation Trust.

### Eligibility criteria {10}

Inclusion criteria:

1. Aged 16 years or older
2. In contact with Secondary Care Mental Health Services (under the care of a consultant psychiatrist)
3. Experiencing current voice-hearing; this will be operationalised by participants having a score of at least 1 on item 1 (How frequently did you hear a voice or voices?) on the Hamilton Programme for Schizophrenic Voices Questionnaire (HPSVQ) [25] – indicating that the participant has experienced at least one episode of voice-hearing in the past week
4. Distressed by hearing voices; operationalised by participants scoring at least 8 out of 16 on the ‘negative impact’ scale of the HPSVQ [25]
5. Meeting DSM-5 research criteria for Schizophrenia Spectrum or Other Psychotic Disorders (assessed by the Structured Clinical Interview for DSM-5 disorders, SCID-5 [26])
6. Willing and able to provide written, informed consent

Exclusion criteria:

1. Established organic cause for distressing voices (e.g. brain disease or injury)
2. Primary diagnosis of substance misuse
3. Currently detained in hospital under a section of the Mental Health Act
4. Have completed a full course (minimum of 16 h) of CBT for psychotic symptoms during the past year
5. Be currently participating, or be confirmed to participate in another interventional study in which they are receiving an intervention which utilises psychological therapy
6. Non-English speaking to the degree that the participant is unable to fully understand and answer assessment questions or give informed consent
7. Severe learning disability – assessed using the Test of Premorbid Functioning – UK (TOPF-UK) [27]
8. Immediate and serious risk to self or other (assessed at the point of referral/eligibility review)

### Who will take informed consent? {26a}

Once a formal referral has been received by the research team, the potential participant will be contacted to discuss the study further and arrange a consent and eligibility meeting with a member of the research team. The potential participant will have a copy of the Participant Information Sheet (PIS) at least 24 h before the consent and eligibility meeting takes place, so they will have time to read the information, discuss it with friends and family, and formulate any questions that they may have.

## Interventions

### Explanation for the choice of comparators {6b}

A three-arm RCT was chosen to enable: (1) any effect of GiVE to be differentiated from two other components of the response to talking therapies (the concerned attention and generic therapeutic effects – both available within SC); and (2) an evaluation of the cost-effectiveness of GiVE – generated by a comparison to the treatments that are usually offered to patients (available within TAU).

### Intervention description {11a}

#### Guided self-help intervention for Voices (GiVE)

The GiVE intervention will be delivered by an AP and will follow a workbook [28] that is based upon the Overcoming Distressing Voices [21] self-help book. Participants will be given a copy of both the workbook and the self-help book at the commencement of therapy by the AP and asked to engage in some level of self-help (homework); this will take the form of reading chapters from the self-help book between therapy sessions and engaging with the suggested activities within the workbook. Participants in the GiVE intervention will also have the opportunity to access the ‘CHOICES’ mobile phone application.

After an introductory session on coping, the intervention will cover three core modules: (1) beliefs about the self, (2) beliefs about voices and (3) relationships. Modules (1) and (2) draw upon psychoeducation and cognitive behavioural strategies to help participants to re-evaluate their negative or unhelpful beliefs related to the self and voices. Module (3) additionally involves work on how to relate to others and voices more assertively. GiVE will be delivered over eight sessions offered over a maximum of 16 weeks. Sessions may be delivered in NHS clinics, participants’ own homes and other community locations as preferred and appropriate. Each session will last for up to 1 h, and sessions will be held weekly where possible (16 weeks are given for the eight sessions to allow for periods of absence, illness, holidays, etc.). Participants in the GiVE arm will continue to receive their usual treatment throughout their participation in the study.

#### Supportive Counselling (SC)

The SC intervention will follow the therapy protocol that was used by APs in the full RCT of AVATAR Therapy for distressing voices [29]. It will be delivered over the same number and duration of sessions as GiVE, with the aim of matching the duration of total therapist contact time across both arms. SC will offer a supportive and non-judgemental space for the discussion of topics and issues determined by the participant. The SC therapy protocol contains specific guidance for the AP on how to respond to participant disclosure of distressing voices in a manner that does not provide specific intervention strategies.

The intervention will involve eight sessions over a maximum of 16 weeks. Each session will last for up to 1 h, and sessions will be held weekly where possible (16 weeks are given for the eight sessions to allow for periods of absence, illness, holidays, etc.). Sessions may be delivered in NHS clinics, participants’ own homes and other community locations as preferred and appropriate. Participants in the SC arm will continue to receive their usual treatment throughout their participation in the study.

The same APs will deliver GiVE and SC in order to minimise the influence of therapist effects; for example, differing levels of therapist competence. They will receive a 5-day training in GiVE and SC (one introductory day, 2 days on GiVE and 2 days on SC), delivered by experienced clinical psychologists and an experienced counselling psychologist, respectively. Weekly clinical supervision will be provided by these clinical/counselling psychologists.

#### Treatment-as-usual

TAU will be provided by the usual care team. TAU should be informed by NICE guidelines (NICE) [4] and typically include medication management and support and monitoring provided by the Adult Mental Health or Early Intervention in Psychosis Services, with individual and family psychological therapies offered occasionally.

### Strategies to improve adherence to interventions {11c}

Therapeutic drift and contamination will be minimised by the use of highly detailed therapy protocols and close supervision of the APs by experienced clinical/counselling psychologists. Adherence to therapy protocols will be assessed through APs completing a checklist at the conclusion of each session. The competence of AP delivery of the interventions will be assessed by the rating of session recordings. All therapy sessions will be audio-recorded (with the participant’s permission) and a random 10% sample rated for competence by independent experts (a clinical psychologist for GiVE and a counselling psychologist for SC). GiVE sessions will be rated using the Cognitive Therapy Rating Scale for Psychosis [30]. SC sessions will be rated using the Counselling Adherence Scale [29].

### Relevant concomitant care permitted or prohibited during the trial {11d}

Participants in all three arms of the trial will be encouraged to engage in, and continue with, existing treatments. Our methodological approach will be to carefully monitor and capture the service contacts received across a range of services in all three arms of the trial using an adapted version of the Client Service Receipt Inventory (CSRI) [31]. Within a future definitive RCT, this will allow us to standardise current practice by providing all referrers with a best practice manual for standard treatment which summarises good practice.

### Outcomes {12}

The assessment of feasibility will include the calculation of the number and proportion of: care co-ordinators (CCs) willing to refer their patients; referred patients found to be eligible; consenting participants who are retained within the study and offer full datasets; non-missing items for each variable; consenting participants within the GiVE and SC arms who reach the point of therapy ‘exposure’ (attended at least six of eight therapy sessions). Feasibility assessment will also measure therapist adherence to therapy and supervision protocols.

### Participant timeline {13}

**Fig. 1:**
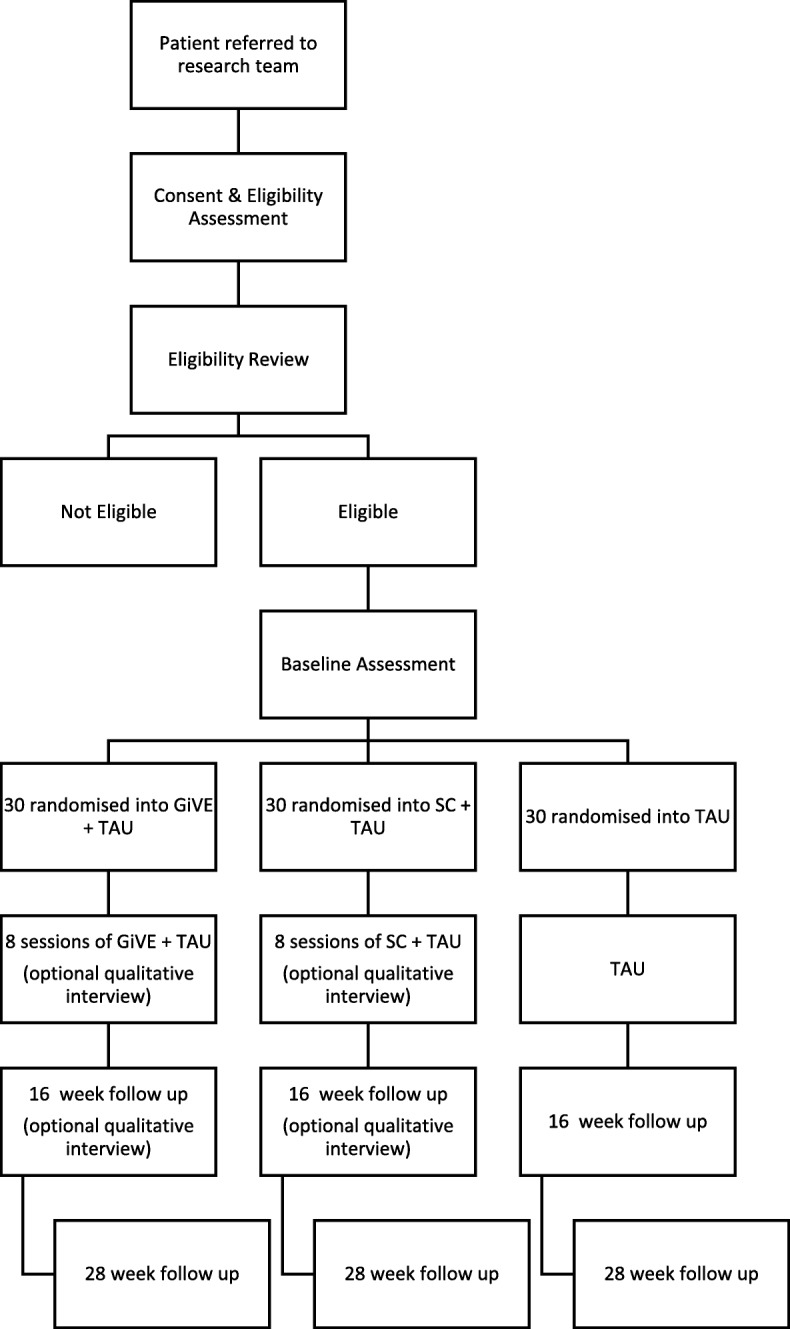
Trial/recruitment flowchart

Figure 1 illustrates the trial/recruitment flowchart.

**Table 1:** Protocol changes

Protocol version	Details of change
Version 2 (2 Jan 2019)	Addition of exclusion criterion for patients currently detained in hospital under a section of the Mental Health Act.
	Addition of Test of Premorbid Functioning as relevant to eligibility assessment of presence of serious learning disability.
	Addition of statement of intent to audio-record participant assessments.
Version 3 (17 Apr 2019)	Reduction of lower age threshold for inclusion from 18 years to 16 years.
	Removal of statement of intent to audio-record participant assessments.
Version 4 (14 Aug 2019)	Addition of questionnaire for referring and non-referring clinicians to explore their decision-making around referring patients with psychoRemoval of statement of intentsis to CBT generally and the GiVE intervention specifically as part of process evaluation.
	Revision of qualitative interview with a subsample of therapy participants to explore their experiences of therapy participation, outcomes, and potential mechanisms of change as part of process evaluation.
Version 5 (25 Sept 2019)	Typographical correction regarding list of participant assessments.

### Sample size {14}

Following recommendations [32] for designing feasibility trials that aim to detect a small-medium standardised effect size (where the definitive trial will be designed with 90% power and two-sided 5% significance), this study aims to recruit 90 patients [33, 34]. Participants will be in contact with Community Secondary Care Adult Mental Health Services or Early Intervention in Psychosis Services at the time of consent, across two sites in the UK: (1) Sussex Partnership NHS Foundation Trust (suburban and rural); and (2) Pennine Care NHS Foundation Trust (suburban and urban).

### Recruitment {15}

Participants will be recruited through referrals from the CCs of psychosis patients (or other appropriate members of the clinical team) in the host sites. When first approached about the study by a research assistant (RA), all CCs will be given a PIS and referral forms. CCs will be asked to share the PIS with any patients who are potentially eligible for the study. If a patient shows an interest in the study and gives their verbal agreement to be contacted by a researcher, the CC will be asked to complete a referral form.

An additional recruitment strategy at the Sussex site will involve the use of the Everyone Counts scheme. Sussex Partnership NHS Foundation Trust has the Everyone Counts scheme in place for consent to contact about research opportunities. Members of the Research and Development Department will contact potentially eligible patients to discuss the study and invite them to take part. Interested patients will be able to contact either the Research and Development Department or the research team directly and enquire about the study. They will be invited to discuss the study with their CC who can then complete a referral form. The research team could support potential participants to discuss the study with their CC if this is needed. An additional strategy will be used in Pennine Care NHS Foundation Trust, whereby patients who have participated in other trials within the Trust and have given consent to be contacted about future studies may be contacted.

## Assignment of interventions: allocation

### Sequence generation {16a} and concealment mechanism {16b}

Participants will be randomly allocated using the Sealed Envelope online service https://www.sealedenvelope.com/. The trial statistician will set up and test the randomisation procedure incorporating stratification by site (Sussex or Pennine) and type of service (Community Mental Health Team or Early Intervention in Psychosis) using random block lengths and 1:1:1 allocation. Participants will be randomly allocated to receive either the study intervention and treatment as usual (GiVE) or the control intervention and treatment as usual (SC) or treatment as usual alone (TAU).

### Implementation {16c}

Consented participants will be randomised by the trial manager using Sealed Envelope. An unblinded member of the research team will then contact the participant by telephone to inform them of their group allocation. APs will be notified by the trial manager about the participants who are allocated GiVE and SC and will be asked to arrange a first appointment, if possible, within 2 weeks following the randomisation. A letter will be sent to all participants to confirm their allocation and details of their next appointment with either the AP (GiVE or SC) or researcher (TAU). This letter will ask participants not to disclose their allocation to the researcher.

## Assignment of interventions: blinding

### Who will be blinded? {17a}

The RAs and the trial statistician will be blind to the allocation sequence. Following the baseline assessment, participants will be randomised using Sealed Envelope and allocated to one of the three trial arms. Only the trial manager will receive this notification and will communicate the allocation to all unblinded members of the research team. Researchers will be blinded to the allocation and will remain blinded for all future assessments with the participants (16-week and 28-week data collection). Measures to maintain blinding will include: (1) participants being reminded at the beginning of each assessment interview to not disclose the group to which they have been allocated; (2) blinded members of the research team being shielded from discussion of participants in forums where the possibility of determining participant allocation could occur; (3) researcher access to electronic health records being restricted; and (4) consideration given to office allocation and all administrative processes of blinded-vs-unblinded members of the research team. ‘Blind’ awareness and education will be promoted throughout the study. To test the success of blinding, the blind RA who assesses each participant will be asked to guess the allocation group for each participant at the end of the final assessment.

### Procedure for unblinding if needed {17b}

Reported breaks in blinding will be recorded. Outcome assessments will be re-blinded by re-allocating ‘blind’ RAs to collect and score study data wherever possible.

## Data collection and management

### Plans for assessment and collection of outcomes {18a}

**Fig. 2:**
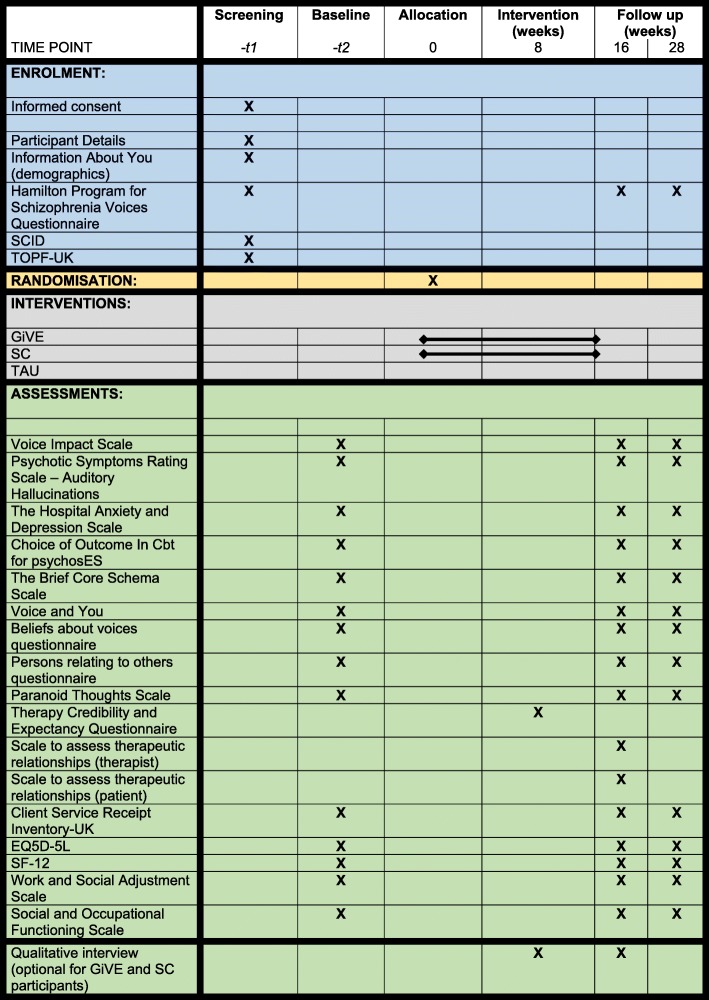
Standard Protocol Items: Recommendations for Interventional Trials (SPIRIT) Figure

See Fig. 2 for details of the assessment at each visit.

#### Screening measures

The assessment of eligibility in relation to the inclusion and exclusion criteria will be supported by the use of the Structured Clinical Interview for DSM-5 Disorders (SCID-5) [26], the Test of Premorbid Functioning–UK (TOPF-UK) [27] and the Hamilton Programme for Schizophrenia Voices Questionnaire (HPSVQ) [25] (each described below).

#### Clinical measures – Primary

The primary outcome for a potential future definitive RCT is the self-reported impact of voice-hearing upon the participants. Two measures will be evaluated as candidates for the primary outcome to be used in a future definitive RCT:

*Psychotic Symptoms Rating Scale (PSYRATS-AH)* [35] Distress Scale – PSYRATS-AH is an 11-item rating scale designed to measure the severity of different dimensions of the voice-hearing experience. Items are grouped together in four factors [36]; distress (negative content, distress and control), frequency (frequency, duration and disruption), attribution (location and origin of voices) and loudness (loudness item only). The ‘distress’ subscale measures the impact that voices have on the individual. The measure has established psychometric properties.

*Hamilton Programme for Schizophrenia Voices Questionnaire (HPSVQ)* [25] Negative Impact Scale – HPSVQ is a 9-item self-report measure of the phenomenology and negative impact of voice-hearing. The ‘negative impact’ subscale has four items rated on a 5-point (0–4) scale and measures the level of distress and impact that voices have on the person. The HPSVQ has been found to have strong concurrent validity (all *r* > 0.80) as well as good internal consistency (all *α* > 0.82) in psychosis patients [37].

#### Clinical measures – Secondary

Secondary outcomes will evaluate: (1) mental health problems commonly experienced by people with psychosis (anxiety and depression (Hospital Anxiety and Depression Scale) [38] and paranoia (Paranoid Thoughts Scale) [39]); (2) variables that have been associated with the impact of voice-hearing (negative beliefs about the self (Self Scale of the Brief Core Schema Scale) [40], negative beliefs about voices (Beliefs About Voices Questionnaire – Revised) [41] and negative relating to voices (Voice and You) [42] and other people (Persons Relating to Others Questionnaire) [43]; and (3) a range of personal and social recovery-oriented outcomes (goals for the outcome of CBTp (CHOICE – short form) [44], the positive impact of voices (Voice Impact Scale) [45], engagement in meaningful activity (Work and Social Adjustment Scale) [46] and social functioning (Social and Occupational Functioning Scale) [47].

#### Process measures

Process measures will evaluate the quality of the therapeutic relationships (Scale to Assess Therapeutic Relationships) [48] and participants’ expectations for therapy (Therapy Credibility and Expectancy Questionnaire) [49].

#### Health economic measures

The *EuroQol 5 dimensions, 5 levels health survey (EQ-5D-5 L)* [50] *–* is a standardised instrument used as a measure of health-related quality of life that can be used in a wide range of health conditions and treatments. The descriptive system comprises five dimensions: mobility, self-care, usual activities, pain/discomfort and anxiety/depression. Each dimension has five levels: no problems, slight problems, moderate problems, severe problems and extreme problems.

The *Client Service Receipt Inventory (CSRI-UK)* [31] *–* is a well-validated adaptable research instrument used in mental health settings to collect information on health, social and voluntary service utilisation, informal care, accommodation, other public services (e.g. police) and private out-of-pocket expenses incurred. Its primary purpose is to allow resource-use patterns to be described and costs to be estimated.

The *Short Form 12 (SF-12)* [51] *–* this generic health survey captures information about functional health and well-being from the patient’s point of view.

### Plans to promote participant retention and complete follow-up {18b}

Efforts will be made to engage all participants in follow-up assessments. RAs will flexibly engage patients, offering appointments at times and locations which best suit participants and offering shorter and split assessment sessions as needed. Participants will be offered reimbursement of £20 per assessment point (i.e. baseline, 16 and 28 weeks) and travel expenses will be made available. Retention rates will be monitored by the trial manager at least weekly and the research team on a monthly basis throughout the trial. The Lived Experience Advisory Panel (LEAP) will be asked to provide consultation regarding retention rates as part of their oversight role.

### Data management {19}

Data collection and analysis will be supervised by the Trial Statistician. The management of the data will be a standing item on the agenda of the monthly meeting of the research team. All members of the research team and any other individuals from collaborating Trusts or Universities involved in collecting, inputting, processing, using and sharing data will have had Information Governance training.

### Confidentiality {27}

The minimum amount of personal information needed to conduct the study will be obtained from participants. Personal and research data will be stored securely on NHS premises. Physical data, such as consent forms, will be locked in filing cabinets on NHS premises accessible only to members of the research team. Electronic data will be stored securely in password-protected or encrypted files on NHS computers accessible only to members of the research team. All research data will be fully anonymised and will be stored separately to personal data. A link file will allow for participant research data to be identified. This link file will be a password-protected file accessible only by members of the trial team within each research site and the trial manager. This file will be securely destroyed following the end of the study. Quantitative and qualitative data will be appropriately aggregated in any publications arising from the trial to protect participant anonymity during and after the trial has ended.

## Statistical methods

### Statistical methods for primary and secondary outcomes {20a}

Analyses will be based on the intention-to-treat approach. All clinical outcomes will be summarised using descriptive statistics at pre-randomisation (T0), at 16 weeks (T1) and at 28 weeks (T2) for each arm (GiVE, SC and TAU) of the study. Next, 95 and 75% confidence intervals [51] will be created for between-group mean differences after adjustment for the baseline scores using analysis of covariance (ANCOVA) for the following comparisons: GiVE vs TAU; GiVE vs SC; and TAU vs SC. There will be no hypothesis testing.

Analysis will be conducted using a linear mixed model with a random effect for individual with fixed effects for treatment group (TAU, GiVE and SC) and time (16 and 28 weeks); baseline score and site will be entered as covariates. Contrasts will be used to compare groups (GiVE vs TAU, GiVE vs SC and TAU vs SC) at T1 and T2. 95 and 75% confidence intervals [25] for between-group mean differences will be created for all estimates of unstandardised effect size.

Standardised (Cohen’s *d*) effect sizes for each outcome will be calculated by dividing the between-group unstandardised effect by the baseline pooled standard deviation. The level of missing data will be summarised for each outcome and reported for each time point.

Two potential primary outcomes – the Distress scale of PSYRATS and the Negative Impact scale of the HPSVQ – are being explored in this feasibility study. In addition to the analyses for all clinical outcomes, the between-groups differences for these two variables will be assessed in the context of:

- Whether the minimal clinically important difference (MCID) is contained within the 95% confidence intervals where:
  - ◦ PSYRATS Distress scale of MCID is a 3-point reduction
  - ◦ HPSVQ Negative Impact scale MCID is a 2-point reduction
- The level of missing data
- Popularity of the outcome in the literature
- Any other relevant information generated by the process evaluation

### Methods for additional analyses {20b}

#### Health economic analysis

An economic evaluation will provide basic information to facilitate the planning of a definitive, multi-centre RCT to assess clinical and cost-effectiveness. The resource requirements and costs of each arm will be investigated using trial details on the delivery of GIVE and SC. Staff time will be costed using national tariffs, inclusive of on-costs and overheads [52]. Participants will be asked to complete the CSRI-UK at 16 weeks and 28 weeks to record all forms of service use (including activity classified as usual care), informal care and out-of-pocket expenses. Information gathered through the CSRI-UK will be cross-checked for accuracy with mental health records. Discrepancies between these records will be recorded and evaluated. Health-related quality of life (HRQoL) will be recorded at baseline and 16 and 28 weeks using both the EQ-5D-5 L and SF-12 for calculation of Quality-adjusted Life Years (QALYs) as the latter may be more sensitive to changes in psychological status. Completeness of data will be assessed. Variability in standard care will be explored, and the use of EQ-5D-5 L and SF-12 compared. Differences in service use between groups will be investigated for indications of potential savings associated with GiVE that might offset the intervention costs. The full range of outcomes will be investigated in a cost consequences framework and a preliminary cost-effectiveness analysis conducted.

#### Process evaluation

The process evaluation will be guided by MRC Guidelines [53] and used to evaluate:

1. Reach of the intervention – the proportion of eligible participants who are referred and who accept the offer of therapy, and responses from referring and non-referring clinicians to a questionnaire designed to explore their decision-making around referring patients with psychosis to CBT generally and the GiVE intervention specifically;
2. Implementation of the intervention – what was delivered in terms of exposure (number of sessions offered and received by participants) and therapist adherence to protocol;
3. How the intervention was delivered – qualitative interviews with the APs delivering GiVE regarding their experience of the training, manuals, supervision and intervention delivery;
4. Context – qualitative interviews with clinicians and managers from referring services, to determine the potential actions, context (team/service/supervisor issues), processes, structures and coherence with standard care which might influence implementation of the GiVE intervention, including sense-making, and potential effort, action, commitment, participation and reflection on implementation of GiVE in routine services;
5. Participant experience – of the research process, randomisation, outcomes, potential mechanisms and mediators, and therapy.

Descriptive data will be used to summarise reach and implementation of the intervention. Qualitative data from the process evaluation interviews will be analysed using thematic analysis [54]; this method will be used to highlight the key themes across interviews in relation to the research questions. These themes will be used to adapt the study design, manuals, training, supervision and intervention delivery where necessary before proceeding to a definitive trial. In addition, the qualitative data will be used to develop both: (1) a system-based logic model for the implementation of the trial and subsequent therapy; and (2) a process-based logic model of the potential mechanisms from therapy experience to outcomes. These will be further refined in the future definitive RCT.

## Oversight and monitoring

### Composition of the Trial Steering Committee (TSC) {5d}

Medical Research Council Guidelines on Good Clinical Practice in Clinical Trials [55] informed the constitution of the TSC, which includes an independent chair, independent experts and lay members.

The scientific integrity of the trial will be overseen by the TSC. The TSC will also serve the functions of a Data Monitoring and Ethics Committee (DMEC), as a combined TSC and DMEC is appropriate within the size and scope of the present feasibility trial, including oversight of the safety and data integrity of the trial.

A separate LEAP will provide Patient and Public Involvement (PPI) oversight of the trial. The LEAP will consult at regular intervals throughout the trial (approximately six meetings in total) and will advise on issues relating to design, delivery, interpretation of findings, dissemination and preparation for a future definitive RCT. Prior to the submission of the grant application for the current study, the LEAP reviewed the differences between the training and role of clinical psychologists (who delivered GiVE in our initial pilot RCT) and APs (who will deliver GiVE in the current study) and emphasised the need for APs to have experience of working with people with psychosis. This became an essential criterion for appointment to the AP post.

### Adverse event (AE) reporting and harms {22}

Any unfavourable and unintended sign, symptom or illness that develops or worsens during the period of the study will be classified as an AE, whether or not it is considered to be related to the study treatment. Adverse events will include: an exacerbation of a pre-existing illness; an increase in the frequency or intensity of a pre-existing episodic event or condition; a condition that is detected after trial intervention administration; and continuous persistent disease or a symptom present at baseline that worsens following administration of the trial treatment – and may be expected or unexpected. Serious adverse events (SAEs) are those considered to be life-threatening, resulting in death, requiring inpatient hospitalisation or prolongation of existing hospitalisation, resulting in significant or persistent incapacity/disability or a birth defect or congenital abnormality. The number (events and individuals) and nature of all events (AEs and SAEs) reported to blind and unblind members of the research team will be recorded.

The period for AE reporting will be following the signing of the study consent form until last follow-up assessment 28 weeks after randomisation. All AEs will be recorded and reviewed by the chief investigator. If an AE is considered to be serious (an SAE), it will be reviewed for causality and expectedness by an independent rater and the sponsor’s representative. The TSC will be informed of the number, nature and review outcome for all SAEs and will be asked to recommend any necessary actions. SAEs will be reported to the NHS Research Ethics Committee as appropriate.

## Dissemination plans {31a}

Trial findings will be disseminated in scientific publications, including feasibility outcomes and process evaluation findings. Findings will be disseminated to participants’ and patient organisations. LEAP members will participate in dissemination including use of social media, producing leaflets for wide distribution and submitting a summary of findings to a non-academic journal. Findings will be presented at patient events and at local, national and international conferences.

## Discussion

CBTp is an evidence-based psychological therapy recommended for psychosis patients within the UK. However, only a minority of patients are offered CBTp. Limited resources have been cited as a prominent reason for this lack of access, leading to calls for shorter forms of CBTp to be developed that can be delivered by briefly trained therapists.

We have responded to this call by developing a brief and targeted form of CBTp for distressing voices – Guided, self-help intervention for Voices (GiVE). We have generated preliminary evidence for the effectiveness of GiVE when delivered by highly trained therapist (clinical psychologists). The next step is to evaluate the experience of stakeholders when GiVE is delivered by briefly trained therapists (APs). The current study will explore the feasibility of conducting this evaluation in the form of a pragmatic, three-arm, parallel-group, superiority 1:1:1 RCT comparing GiVE to an active control intervention (SC) and TAU.

CBTp offered in these less resource-intensive forms has the potential to generate benefits for: (1) individual patients (reduced distress and enhanced recovery and enhanced quality of life); (2) service-level patient benefit (increased access to evidence-based psychological therapies); and (3) economic benefits to the NHS (in terms of the reduced use of mental health inpatient services).

## Trial status

Recruitment to the trial commenced in February 2019 (study protocol – version 1, dated 5 November 2018). At present, recruitment and data collection will continue until July 2020.

## Supplementary information

**Additional file 1:** Participant Information Sheet (Patient participants).

**Additional file 2:** Consent Form (Patient participants).

## Data Availability

Not currently applicable. The fully anonymised datasets generated and/or analysed (and associated statistical code) during the current study will be available from the corresponding author on reasonable request following the publication of results.

## Abbreviations

AP: Assistant psychologist
CBTp: Cognitive Behaviour Therapy for psychosis
CC: Care co-ordinator
CHOICE: CHoice of Outcome In Cbt for psychosEs
CSRI: Client Service Receipt Inventory
DMEC: Data Monitoring and Ethics Committee
DSM: Diagnostic and Statistical Manual for Mental Disorders
GiVE: Guided self-help CBT intervention for Voices
HPSVQ: Hamilton Programme for Schizophrenic Voices Questionnaire
HRQoL: Health-related quality of life
MCID: Minimum clinically important difference
MRC: Medical Research Council
NHS: National Health Service
NICE: National Institute for Health and Care Excellence
NIHR: National Institute for Health Research
PIS: Participant Information Sheet
PSYRATS: Psychotic Symptoms Rating Scale
QALYs: Quality-adjusted Life Years
RA: Research assistant
RCT: Randomised controlled trial
SC: Supportive Counselling
SCID-5: Structured Clinical Interview for DSM-5 disorders
SF-12: Short Form12 health survey
TAU: Treatment as usual
TOPF-UK: Test of Premorbid Functioning–UK
TSC: Trial Steering Committee

## Footnotes

1 This assessment will only be offered to participants who reach the 28 weeks’ post-randomization milestone before the end of the data collection period.
