# Supplementary material for: Increasing access to CBT for psychosis patients: a feasibility, randomised controlled trial evaluating brief, targeted CBT for distressing voices delivered by assistant psychologists (GiVE2)
Source: Trials. 2020 Apr 1;21:302. doi: 10.1186/s13063-020-4181-y (PMC7110645; doi:10.1186/s13063-020-4181-y)
Supplement: Supplementary file 1 — Additional file 1: Participant Information Sheet (Patient participants). [file 13063_2020_4181_MOESM1_ESM.docx]

INSERT NHS TRUST DETAILS HERE

**Participant Information Sheet**

**(Patient participants, Version 5.0 24/09/19)**

**GiVE2: Increasing access to CBT for psychosis patients: a feasibility randomized controlled trial evaluating brief, targeted CBT for distressing voices delivered by Assistant Psychologists**

**This project is funded by the National Institute for Health Research for Patient Benefit (project number PB-PG-0317-20029)**

You have been given this information sheet because you are being invited to take part in a research project. It is up to you to decide whether or not you want to take part. Before you decide, it is important that you know about the project. This information sheet will tell you about why the project is being done and what will happen if you decide to take part. Please take as much time as you need to read and understand this information: you can talk about it with other people if you want. If there is anything you don’t understand you can speak to a member of the GiVE2 team. You will find the team’s contact details at the end of this information sheet.

**Why is the project being done?**

The National Institute for Health & Care Excellence (NICE) recommend Cognitive Behaviour Therapy (CBT) as one of the best treatments for psychosis. But only 10% of people with psychosis have the chance to receive CBT. CBT is scarce because it can be quite long and needs to be delivered by highly trained therapists. We want to see if a shorter version of CBT that is delivered by therapists with less training will be helpful for people who hear voices.

The purpose of this study is to find out if a shorter version of CBT delivered by briefly trained therapists can be helpful for people who hear distressing voices. We will evaluate this form of CBT by comparing it to two control groups – one group who will receive supportive counselling and another group who will receive no additional interventions.

Findings of this study would provide helpful information and inform future research into using CBT for people who experience distressing voice hearing.

**Why have I been invited to take part?**

You have been invited to take part in the study because we understand that you are currently distressed by hearing voices. Ninety people will take part in the study; thirty will receive CBT in addition to their usual care; thirty will be in a control group who receive a supportive counselling intervention in addition to their usual care; and thirty will be in another control group who receive only their usual care.

**Do I have to take part?**

No, it is up to you to decide whether or not you want to take part. Even if you do decide to take part, you can change your mind at any time without having to give a reason. If you decide not to take part, or to stop taking part, this will not affect the care you receive from your care team.

**What will happen if I decide to take part?**

If you decide to take part, you will meet with a GiVE2 research assistant to complete an assessment to see if you are eligible to take part in the study. The research assistant will be independent from your care team. In the assessment the research assistant will ask you to complete a consent form, a questionnaire about your experience of voice hearing and a structured clinical interview.

**Are there any reasons why I wouldn't be able to take part?**

You cannot take part if you are under the age of 16, hear voices due to an organic cause (e.g. a brain disease such as dementia, or a brain injury) and/or have a primary diagnosis of substance misuse. Receiving this participant information sheet doesn't necessarily mean you will be able to take part in this study. The eligibility assessment will need to be carried out to determine if this study is right for you.

**What will happen if I am eligible to take part?**

If you are eligible to participate within the study, you will be asked to complete a further assessment with a research assistant. This assessment will require you to complete some more questionnaires. After this assessment, you will be randomly allocated to one of the three groups of the study; one group will receive CBT in addition to their usual care; another group will receive a supportive counselling intervention in addition to their usual care; and another group will receive only their usual care. The allocation to different groups is completely random, and no one in the research team has control over the allocation of participants.

Sixteen weeks later you will be invited to meet with a member of the research team to repeat the completion of the questionnaires.

A further twelve weeks later you may be invited to meet with a member of the research team for a final time to repeat the completion of the measures.

To compensate you for your time, you will be paid £20 per assessment that you complete. The cost of travelling to the assessments can be repaid.

If you are allocated to receive either CBT or supportive counselling, you may also be invited to complete up to two interviews that will involve talking about your experiences of the therapy – what was good, what wasn’t so good, what changes should be made, etc. The interviews will be audio recorded. Taking part in these interviews is optional, and the invitations can be declined.

**What would happen if I was allocated to receive therapy?**

If you are allocated to receive one of the therapies (either CBT or supportive counselling), a therapist will contact you to arrange the therapy sessions. The therapy will involve eight weekly sessions with a therapist, and each session will last for up to an hour. You will still be able to continue with your usual care in addition to receiving the therapy.

If you are allocated to receive CBT, this therapy will help you to explore different ways of thinking about yourself and your voices, as well as your relationships. The therapy will also help you to learn about different ways to respond to and manage distressing voices.

If you are allocated to receive the supportive counselling intervention, this therapy will help you to explore general issues and problems related to your mental health.

We will ask your permission to audio-record all of the therapy sessions. The recordings of the sessions will be securely stored and used by the therapists and other members of the research team to ensure that the therapy is being delivered as planned.

**What would happen if I was not allocated to receive therapy?**

If you are allocated to the group who will not be receiving therapy, you will continue to receive your usual care from your care team. Taking part in this study will have no effect on your current treatment provision.

**Where would I have to go?**

The meetings with a research assistant will take place in a convenient location for you. This may be your GP surgery, the place where your care team is based or your home. For those allocated to receive therapy, the therapy sessions would take place at the base used by your local mental health service. If it is not possible for you to travel to this base, therapy may be offered at your home.

**What are the possible risks of taking part?**

The CBT being evaluated within this study is being delivered in a novel way. For this reason, we do not know whether it will be helpful to patients, and we do not know if it will be helpful for you. By taking part in the study you will be helping us to learn if CBT is helpful when delivered in this way, and this will help mental health services when they are planning what therapies they offer.

Your care team including your Care Co-ordinator or Lead Practitioner would know that you were taking part in the study. For those offered the therapy, you would still receive your usual care as well.

Talking about experiences of hearing voices can be helpful, though it can also sometimes feel difficult or distressing. The therapists will be trained in helping people with distressing voices and would help you cope with any temporary increases in distress, should this occur. The therapists will be supervised by experienced Clinical Psychologists. You would also be free to access help from your care team, should you wish, and to drop out of the therapy if you wished.

The study will involve giving up some of your time to take part. To reimburse you for your time we will pay you £20 per assessment you complete (baseline and two follow up assessments). All participants can potentially earn £60 over the course of the study.

**What are the possible benefits of taking part?**

We hope that the CBT will be helpful, but we can’t guarantee this. The information we find out from this research will help provide helpful information about whether CBT delivered by briefly trained therapists is helpful for people who hear distressing voices. This will help mental health services to make decisions about what therapies should be provided for people who hear voices.

**What happens when the research stops?**

When the research project is finished, all participants will continue to receive their usual care from their care team.

**What happens if something goes wrong?**

It is very unlikely that there will be any adverse events associated with taking part in this research study. However, if you are harmed by taking part in this research study, there are no special compensation arrangements. If you are harmed by someone’s negligence, then you may have grounds for a legal action, but you may have to pay for it.

If you would like to make a complaint regarding your treatment and participation in this study please contact:

**Tanya Telling**

Research and Development Department

Sussex Partnership NHS Foundation Trust

01273 242034

[Tanya.telling@sussexpartnership.nhs.uk](mailto:Tanya.telling@sussexpartnership.nhs.uk).

If you would like to speak to someone independent of the GiVE2 team, for more information or if something goes wrong, you can speak to your local PALS (Patient Advice and Liaison Service).

**[Local PALS contact details]**

**Will my taking part in this project be kept confidential?**

All information collected as part of this research including consent forms, questionnaires, notes and recordings of interviews will be kept in a locked filing cabinet and secure computer systems at NHS sites. Data will be transferred between sites securely to ensure no-one outside the research team is able to access it. Any information from or about you will have your name, address and any other identifying features removed so that you cannot be recognised from it. This means that your anonymity will be preserved at all times during and after the study time period. All data will be destroyed 10 years after the study has been completed in line with NHS research policy.

If you are in one of the therapy groups, we will audio record the sessions you have with your therapist with your permission. This is because we want to check that the therapy is being carried out in the way that we expect. All recordings will be stored securely and anyone listening to the tapes will sign a declaration of confidentiality.

The only people that will know about your participation in this study will be the research team, your care team, and anyone else that you choose to tell. Although your care team (e.g. your GP or care coordinator/lead practitioner) will be aware that you are taking part in the study, they will not be told any of the information that you share during your participation in the research study e.g. the results of the questionnaires. We will only break this confidentiality if you mention something that puts either yourself or others at risk. Under these circumstances we will have to pass on this information to your care team.

Sussex Partnership NHS Foundation Trust is the sponsor for this study based in the United Kingdom. We will be using information from you in order to undertake this study and will act as the data controller for this study. This means that we are responsible for looking after your information and using it properly. Sussex Partnership NHS Foundation Trust will keep identifiable information about you for 10 years after the study has finished.

Your rights to access, change or move your information are limited, as we need to manage your information in specific ways in order for the research to be reliable and accurate. If you withdraw from the study, we will keep the information about you that we have already obtained. To safeguard your rights, we will use the minimum personally-identifiable information possible.

You can find out more about how we use your information by contacting Researchgovernance@sussexpartnership.nhs.uk

[Sussex Partnership NHS Foundation Trust or INSERT NHS] will use your name, NHS number and contact details to contact you about the research study, and make sure that relevant information about the study is recorded for your care, and to oversee the quality of the study. Individuals from Sussex Partnership NHS Trust and regulatory organisations may look at your medical and research records to check the accuracy of the research study. *[Insert NHS site] will pass these details to Sussex Partnership NHS Trust along with the information collected from you and your medical records.* The only people in Sussex Partnership NHS Trust who will have access to information that identifies you will be people who need to contact you for research purposes and to analyse the research data.

[Sussex Partnership NHS Foundation Trust or INSERT NHS] will keep identifiable information about you from this study for 10 years.

**What will happen to the result of the research?**

The results of the study will be written-up as a report that will hopefully be published in a psychology journal. These results may also be presented at academic conferences. You will also be asked if you would like a copy of the results when completing the consent form.

**Who is organising and paying for the research?**

The research is being paid for by the Department of Health’s National Institute of Health Research (NIHR) and sponsored by Sussex Partnership NHS Foundation Trust. The research is being carried out by researchers from Sussex Partnership NHS Foundation Trust, Pennine Care NHS Foundation Trust, University of Sussex and the University of Manchester.

**Who has approved the research?**

Research projects like this one can’t go ahead without being approved by an NHS Research Ethics Committee. The Ethics Committee checks that the risks associated with the study have been reduced to a minimum and balanced against potential benefits. They also check that patients have been given enough information to make an informed choice about whether or not to take part. This study had been considered and approved by London-Surrey Research Ethics Committee.

**Where can I get more information?**

For general information about taking part in research you can contact your local NHS Trusts’ research and development department:

**[Local R&D contact details]**

If you need further information about this specific project please contact a member of the GiVE2 team. You can contact the team at any time using the following email address:

**[Local team email address]**

If you would prefer to contact the team by phone, the names and telephone numbers of some GiVE2 team members are given below. They can be reached during office hours, Monday to Friday

Local Research Assistant Clio Berry

Research Assistant Trial Manager

Telephone number 0300 304 0088 / 07738 757570

**Next Steps:**

If you are interested in taking part in the study, please allow yourself at least 24 hours to consider your decision before contacting the research team or before asking for a member of the research team to contact you. This is to ensure that you have had time to consider your decision.

**If you would like to take part in the study, or to find out further information please call**

Local Research Assistant Clio Berry

Research Assistant Trial Manager

Telephone number 0300 304 0088 / 07738 757570

Thank you for taking the time to read this information sheet.
