# Supplementary material for: Increasing access to CBT for psychosis patients: a feasibility, randomised controlled trial evaluating brief, targeted CBT for distressing voices delivered by assistant psychologists (GiVE2)
Source: Trials. 2020 Apr 1;21:302. doi: 10.1186/s13063-020-4181-y (PMC7110645; doi:10.1186/s13063-020-4181-y)
Supplement: Supplementary file 2 — Additional file 2: Consent Form (Patient participants). [file 13063_2020_4181_MOESM2_ESM.docx]

**Consent Form (Patient Participants)**

**Study Title:** GiVE2: Guided self-help CBT intervention for VoicEs.

**Chief Investigator:** Mark Hayward

**Participant Identification Number:**

| 1. | I confirm that I have read and understood the Participant Information Sheet for the above study, and have had the opportunity to ask questions | |  |
| --- | --- | --- | --- |
| 2. | I understand that my participation is voluntary and that I am free to withdraw at any time, without giving any reason, and without my medical care or legal rights being affected | |  |
| 3. | I understand that if I choose to withdraw that any data provided up to that point will be kept by the research team | |  |
| 4. | I give my permission for my anonymized data to be shared with others outside the research team for research purposes | |  |
| 5. | I understand that should I disclose anything that presents new risk to either myself or others then Trust risk procedures will be followed, which may involve the release of my personal information | |  |
| 6. | I give permission for my GP and/or Lead Practitioner to be informed of my participation in the above study | |  |
| 7. | I give permission, if I receive therapy, for the therapy sessions to be recorded, as a means of checking the therapist competency | |  |
| 8. | I agree to complete an interview discussing my experiences of the therapy if I receive it | |  |
| 9. | I understand that relevant sections of my medical notes and data collected during the study may be looked at by regulatory authorities or from the NHS Trust, where it is relevant to my taking part in this research. I give permission for these individuals to have access to my records. | |  |
| 10. | I give my permission for members of the research team to access my medical records for research purposes, only when the needed information isn’t available from another source. | |  |
| 11. | I agree to take part in the above study | |  |
| 12 | I give permission for my contact details and information to be kept so I might be invited to participate in future research | |  |
| Name of Participant: | | | |
| Participant Signature: | | Date: | |
| Name of Researcher: | | | |
| Researcher Signature: | | Date: | |
